# Aluminum-Induced Alterations in Purinergic System Parameters of BV-2 Brain Microglial Cells

**DOI:** 10.1155/2021/2695490

**Published:** 2021-01-12

**Authors:** Charles Elias Assmann, Vitor Bastianello Mostardeiro, Grazielle Castagna Cezimbra Weis, Karine Paula Reichert, Audrei de Oliveira Alves, Vanessa Valéria Miron, Margarete Dulce Bagatini, Taís Vidal Palma, Cinthia Melazzo de Andrade, Micheli Mainardi Pillat, Fabiano Barbosa Carvalho, Cristina Ruedell Reschke, Ivana Beatrice Mânica da Cruz, Maria Rosa Chitolina Schetinger, Vera Maria Melchiors Morsch

**Affiliations:** ^1^Postgraduate Program in Biological Sciences, Toxicological Biochemistry, Department of Biochemistry and Molecular Biology, Federal University of Santa Maria, Santa Maria, RS, Brazil; ^2^Postgraduate Program in Food Science and Technology, Department of Food Science and Technology, Federal University of Santa Maria, Santa Maria, RS, Brazil; ^3^Postgraduate Program in Pharmacology, Department of Physiology and Pharmacology, Federal University of Santa Maria, Santa Maria, RS, Brazil; ^4^Postgraduate Program in Biomedical Sciences, Federal University of Fronteira Sul, Chapecó, SC, Brazil; ^5^Department of Microbiology and Parasitology, Federal University of Santa Maria, Santa Maria, RS, Brazil; ^6^Federal University of Health Sciences of Porto Alegre, Porto Alegre, RS, Brazil; ^7^School of Pharmacy and Biomolecular Sciences, Royal College of Surgeons in Ireland, Dublin, Ireland; ^8^FutureNeuro Research Centre, Dublin, Ireland; ^9^Postgraduate Program in Gerontology, Federal University of Santa Maria, Santa Maria, RS, Brazil

## Abstract

Aluminum (Al) is ubiquitously present in the environment and known to be a neurotoxin for humans. The trivalent free Al anion (Al^3+^) can cross the blood-brain barrier (BBB), accumulate in the brain, and elicit harmful effects to the central nervous system (CNS) cells. Thus, evidence has suggested that Al increases the risk of developing neurodegenerative diseases, particularly Alzheimer's disease (AD). Purinergic signaling has been shown to play a role in several neurological conditions as it can modulate the functioning of several cell types, such as microglial cells, the main resident immune cells of the CNS. However, Al effects on microglial cells and the role of the purinergic system remain elusive. Based on this background, this study is aimed at assessing the modulation of Al on purinergic system parameters of microglial cells. An *in vitro* study was performed using brain microglial cells exposed to Al chloride (AlCl_3_) and lipopolysaccharide (LPS) for 96 h. The uptake of Al, metabolism of nucleotides (ATP, ADP, and AMP) and nucleoside (adenosine), and the gene expression and protein density of purinoceptors were investigated. The results showed that both Al and LPS increased the breakdown of adenosine, whereas they decreased nucleotide hydrolysis. Furthermore, the findings revealed that both Al and LPS triggered an increase in gene expression and protein density of P2X7R and A2AR receptors, whereas reduced the A1R receptor expression and density. Taken together, the results showed that Al and LPS altered the setup of the purinergic system of microglial cells. Thus, this study provides new insights into the involvement of the purinergic system in the mechanisms underlying Al toxicity in microglial cells.

## 1. Introduction

The prevalence of chronic neurodegenerative disorders, such as Alzheimer's disease (AD), is growing considerably with the rise of global life expectancy. The gradual decline of motor and/or cognitive capacities associated with these morbidities is of major concern; however, the underlying mechanisms are still not fully understood [1, 2]. Evidence has pointed out that some environmental factors, such as aluminum (Al), could be an etiological cause and associated with a higher risk of developing AD [3, 4]. Al is a lightweight metal ubiquitously found in the environment, resulting in a nearly unavoidable contact of humans to this element [5]. Human exposure to this metal can occur by several routes, including drinking water, foods, occupational exposure, and vaccines, among others [5, 6].

Its highly toxic form, the trivalent free Al ion (Al^3+^), can cross the blood-brain barrier (BBB), alter membrane function, and accumulate in the human brain throughout life [6–9]. Literature data have indicated that Al is a neurotoxic metal that may lead to alterations associated with neurodegeneration and brain aging, among others, characteristics also found in AD [3, 9–11]. Moreover, recent evidence using a model of neural progenitor cells (NPCs) have suggested that Al^3+^ may also affect neurogenesis [12] and purinergic signaling [13].

The purinergic signaling system includes a cascade of extracellular nucleotides- and nucleoside-catalyzing enzymes that participate in the metabolism of ATP (adenosine triphosphate) to ADP (adenosine diphosphate) and/or AMP (adenosine monophosphate) (NTPDase/CD39, nucleoside triphosphate diphosphohydrolase), followed by the conversion of AMP to adenosine (5′-NT/CD73, 5′-nucleotidase) and of adenosine to inosine (ADA, adenosine deaminase) [14–17]. Moreover, ATP and adenosine can operate as signaling molecules through their binding to specific purinergic receptors. P1 adenosine receptors (A1, A2A, A2B, and A3), which are all G protein-coupled receptors, are selective to adenosine. Purine nucleotides, such as ATP and ADP, and pyrimidine nucleotides, such as UTP (uridine triphosphate) and UDP (uridine diphosphate), act on P2 receptors, which are subdivided into P2X ionotropic receptors (P2X1-7) and P2Y receptors (P2Y1/2/4/6/11/12/13/14) which are coupled to G proteins [18–20].

Purinergic signaling may operate in a wide range of biological functions including neurotransmission and neuroinflammation and also in disease conditions [21–23]. Particularly in the central nervous system (CNS), this signaling pathway may also orchestrate immune cell responses, including microglial activation and the release of inflammatory cytokines, among others [22, 24]. Microglia comprise the main cells of the neuroimmune system acting in the clearance of noxious stimuli and limiting tissue damage [25, 26] and exhibit fundamentally all the repertoire of components of the purinergic system [22, 24].

However, mechanisms of Al toxicity in the brain related to the purinergic system, especially on microglial cells, remain elusive and it is essential to understand the role of this metal in the neuroinflammatory responses and neurodegenerative conditions. In this sense, this study is aimed at primarily investigating whether the exposure to Al (in the AlCl_3_ form) could alter some purinergic system parameters using an *in vitro* model of microglial cells.

## 2. Materials and Methods

### 2.1. Chemicals and Reagents

This investigation used chemicals and reagents of analytical grade obtained from Sigma-Aldrich Inc. (St. Louis, MO, USA) and Merck KGaA (Darmstadt, Germany). All other reagents otherwise not stated were of chemical purity. Materials used in cell culture procedures were acquired from Kasvi (São José dos Pinhais, PR, Brazil), Corning Inc. (Corning, NY, USA), and Vitrocell Embriolife (Campinas, SP, Brazil). Western blot and molecular biology reagents were purchased from Bio-Rad Laboratories (Hercules, CA, USA), Merck KGaA, and Sigma-Aldrich Inc. All measurement analyses were carried out using a SpectraMax® i3 Multimode Plate Reader (Molecular Devices, Sunnyvale, CA, USA). The fluorescent images were captured using an Olympus Fluorescent Microscope (Fluoview FV101, Olympus, Tokyo, Japan).

### 2.2. Al

For this research, Al^3+^ in the chloride form (AlCl_3_; molecular weight 133.34 g mol^−1^; 99% purity) was purchased from Sigma-Aldrich Inc. All laboratory materials (flasks, plates, tips, tubes, etc.) and glassware were immersed in a 10% HNO_3_/ethanol (*v*/*v*) solution for 48 h and then washed with Milli-Q® ultrapure water to avoid external contamination by the metal. All solutions were prepared using decontaminated materials and Milli-Q® ultrapure water. All laboratory and cell culture protocols were performed in a clean workbench to avoid contamination by external Al sources.

### 2.3. Experimental Design of Cell Culture Protocols and Exposure to Toxicants

This *in vitro* study used the mouse brain BV-2 microglial cell line, purchased from the Rio de Janeiro Cell Bank (BCRJ, Rio de Janeiro, RJ, Brazil). The cells were cultured using *Roswell Park Memorial Institute* (RPMI) 1640 medium (4500 mg/L glucose, 1500 mg/L sodium bicarbonate, 2 mM L-glutamine, and 1 mM sodium pyruvate), added with fetal bovine serum (FBS) to a final concentration of 10% and supplemented with 1% penicillin/streptomycin (10.000 U/mL; 10 mg/mL). The cells were grown in standard conditions by using a humidified and controlled atmosphere of 5% carbon dioxide (CO_2_) at 37°C.

The cells were treated with an increasing concentration-effect curve of the trivalent free Al ion (Al^3+^) in the form of Al chloride (AlCl_3_), consisting of 1, 5, 10, 50, 100, 500, and 1000 *μ*M of AlCl_3_, based on a previous work [27]. The control group (C) consisted of cells that received only the culture medium. Lipopolysaccharide (LPS) at the concentration of 1 *μ*g/mL was used as a positive inflammatory control since it has been suggested that this agent causes neuroinflammatory states [28, 29]. For culture protocols, cells were grown in 6-well plates at the concentration of 1 × 10^5^ cells/mL for 96 h to investigate the subchronic effects of Al and LPS exposure.

### 2.4. Al Staining

Lumogallion (Santa Cruz Biotechnology, Inc., Dallas, TX, USA) is a reagent that can be used for the detection of Al in both tissues and cells. In brief, for this assay, cells were initially prepared on poly-lysine-coated slides and then incubated in the dark at 37°C for 24 h with Lumogallion (100 *μ*M). Then, slides were washed with ultrapure water, air-dried, and fixed in paraformaldehyde (PFA, 4%) at room temperature for 15 min and washed again. DAPI (4′,6-diamidino-2-phenylindole) reagent (0.3 mg/mL, Sigma-Aldrich Inc.) was used to stain cell nuclei. Labeling with DAPI was performed protected from light during 5 min at room temperature, and subsequently, the staining solution was removed, cells were washed, and coverslips were placed on slides, following similar procedures described before [30, 31]. Finally, images were taken using an Olympus Fluorescent Microscope.

### 2.5. Purinergic System Enzyme Activities

#### 2.5.1. NTPDase and 5′-NT Activities

The release of inorganic phosphate was employed to determine the enzymatic activities of NTPDase [32] and 5′-NT [33], similarly to guidelines published before. Briefly, cells were initially suspended in saline (NaCl, 0.9%), and 20 *μ*L of samples was added to the reaction mixture of each enzyme and preincubated at 37°C for 10 min. The enzymatic reaction was initiated by adding the specific substrates for each enzyme: ATP and ADP for NTPDase and AMP for 5′-NT.

The reactions were stopped by the addition of trichloroacetic acid (TCA, 10%), and the released inorganic phosphate due to ATP, ADP, and AMP hydrolysis was determined by using malachite green as the colorimetric reagent. A standard curve was prepared with KH_2_PO_4_, and absorbance was read at 630 nm. Controls were performed to correct for nonenzymatic hydrolysis. Enzyme-specific activities were reported as nmol of Pi released per min per mg of protein.

#### 2.5.2. ADA Activity

ADA activity was performed based on the measurement of ammonia produced when this enzyme acts in the excess of adenosine, following a previously published method [34]. In brief, 50 *μ*L of cell suspension reacted with 21 mmol/L of adenosine (pH 6.5) at 37°C for 60 min. After the incubation period, the reaction was stopped by the addition of 167.8 mM sodium nitroprusside, 106.2 mM phenol, and a sodium hypochlorite solution. The amount of ammonia produced was quantified at 620 nm, and the results were expressed as U Ado (adenosine) per mg of protein.

### 2.6. Purinergic System Receptors

#### 2.6.1. Gene Expression

The gene expression modulation of the purinergic receptors P2X7R (*P2rx7*), A2AR (*Adora2a*), and A1R (*Adora1*) was carried out by real-time quantitative polymerase chain reaction (RT-qPCR) analysis, based on a previous report [35]. Briefly, RNA was initially obtained from samples with TRIzol™ reagent (Invitrogen™), quantified spectrophotometrically, and reversely transcribed into cDNA with the iScript™ cDNA synthesis kit (Bio-Rad Laboratories), by the addition to each sample of 1 *μ*L of iScript reverse transcriptase and 4 *μ*L of the iScript Mix. Steps of the reaction were as follows: 25°C for 5 min, 42°C for 30 min, 85°C for 5 min, and a final step of 5°C for 60 min, performed using a thermal cycler equipment.

The RT-qPCR reaction was performed using 19 *μ*L of a mix containing the iTaq Universal SYBR Green Supermix (Bio-Rad Laboratories) and 1 *μ*L of the cDNA sample. The parameters used were a holding step of 3 min at 95°C, followed by a cycling step of 40 cycles at 95°C for 10 s, 60°C for 30 s, and last a melting step with a melting curve of 65°C to 95°C with an increase of 0.5°C for 5 s. The relative expression of each gene was represented as the fold expression compared to the control group and calculated by using the comparative *^ΔΔ^*CT value. The *β*-actin (*Actb*) gene was used as the internal control to normalize gene expression. The forward and reverse sequences of oligos (5′-3′) used for each gene were as follows: *β*-actin (F): CCGTAAAGACCTCTATGCCAAC; *β*-actin (R): AGGAGCCAGAGCAGTAATCT; P2X7R (F): CTTTGCTTTGGTGAGCGATAAG; P2X7R (R): CACCTCTGCTATGCCTTTGA; A1R (F): GGCCATAAAGTCCTTGGGAAT; A1R (R): CAGGAAGTTCAGGGCAAGAA; A2AR (F): TCACGTCTCAGGATTGAGTTTAG; A2AR (R): CCCGAAGGAAAGGCAGTAG.

#### 2.6.2. Protein Density

Protein density by Western blot analysis of the P2X7R, A2AR, and A1R receptors was performed based on a prior work [36]. In brief, cells were initially homogenized in ice-cold radioimmunoprecipitation assay (RIPA) buffer added with 1 mM phosphatase and protease inhibitors and centrifuged at 12,000 rpm at 4°C for 10 min. Subsequently, samples were separated using sodium dodecyl sulfate-polyacrylamide gel electrophoresis (SDS-PAGE) and transferred to Immun-Blot® PVDF membranes (Bio-Rad Laboratories). After blocking, membranes were incubated overnight at 4°C with the primary antibodies: P2X7R (dilution 1 : 500), A2AR (dilution 1 : 800), and A1R (dilution 1 : 500), all obtained from Santa Cruz Biotechnology, Inc. After this step, membranes were washed with Tris-buffered saline (pH 7.6) with 0.1% Tween 20 (TBST) and further incubated with anti-rabbit or anti-mouse secondary antibodies (dilution 1 : 10.000, Santa Cruz Biotechnology, Inc.) at room temperature for 90 min. The membranes were washed again, incubated with an enhanced chemifluorescent substrate (Immobilon® Forte Western HRP Substrate, Merck KGaA), and analyzed with a ChemiDoc Imaging System (Bio-Rad Laboratories). As a control for protein concentration, membranes were reprobed and tested for *β*-actin immunoreactivity (dilution 1 : 1000, Santa Cruz Biotechnology, Inc.).

### 2.7. Protein Determination

The protein in samples was determined using the Coomassie Blue reagent following the method previously described and using serum albumin as standard [37]. The protein of samples (mg/mL) was adjusted according to each assay.

### 2.8. Statistical Analysis

The results were compared by one-way analysis of variance (ANOVA) followed by Tukey's *post hoc* test and presented as the mean ± SD. The GraphPad Prism software version 6 (GraphPad Software, Inc.; La Jolla, CA, USA) was used to perform statistical analysis. The results were considered statistically significant when *p* < 0.05. All experiments were carried out in triplicate.

## 3. Results

### 3.1. Al Can Be Tracked by Lumogallion Fluorescent Probe in BV-2 Microglial Cells

**Figure 1:**
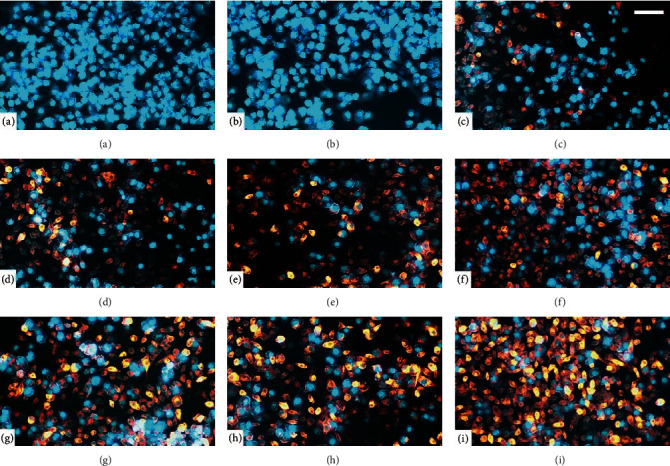
Representative fluorescence microscopy of microglial cells stained with DAPI (blue, cell nuclei) and Lumogallion probe (orange) to track uptake of Al: (a) control; (b) lipopolysaccharide (LPS, 1 *μ*g/mL); (c) AlCl_3_ (1 *μ*M); (d) AlCl_3_ (5 *μ*M); (e) AlCl_3_ (10 *μ*M); (f) AlCl_3_ (50 *μ*M); (g) AlCl_3_ (100 *μ*M); (h) AlCl_3_ (500 *μ*M); (i) AlCl_3_ (1000 *μ*M). Magnification = 200x. Scale bar = 20 *μ*m. *n* = 3.

To track the possible uptake of Al by cells, we used Lumogallion staining and demonstrative images are shown in Figure 1. Control cells (Figure 1(a)) and cells cultured with LPS (Figure 1(b)), both in the absence of Al, only showed blue fluorescence produced by DAPI, which is used to indicate cell nuclei. In this case, when images taken with DAPI and Lumogallion reagent were overlaid, only blue fluorescence was emitted. However, cells cultured in the presence of AlCl_3_ at the concentrations of 1-1000 *μ*M (Figure 1(c)–1(i)) showed an orange labeling representative of Lumogallion staining, and overlaid images also revealed a blue fluorescence related to DAPI nuclei staining. Thus, the free Al ion can be internalized by microglial cells as suggested by the orange concentration-dependent intensity tracked by Lumogallion staining.

### 3.2. Al and LPS Alter the Activity of Purinergic System Ectoenzymes

**Figure 2:**
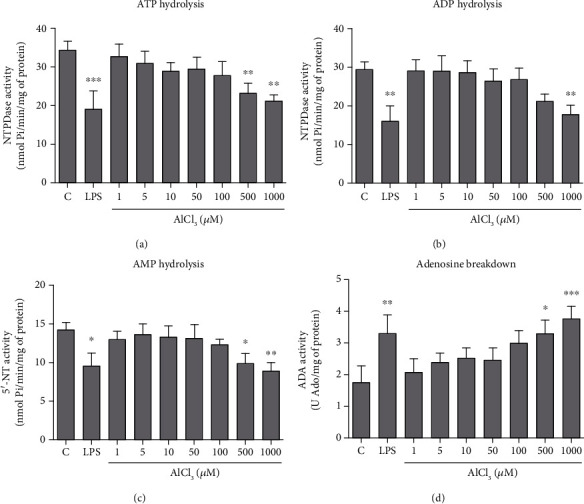
Purinergic enzyme activities of microglial cells treated with LPS (1 *μ*g/mL) and increasing concentrations of AlCl_3_ (1–1000 *μ*M) for 96 h. NTPDase activity using (a) ATP and (b) ADP as substrates, (c) 5′-NT activity using AMP as substrate, (d) ADA activity using adenosine as substrate. C = control group; LPS = lipopolysaccharide. Values are expressed as mean ± SD (*n* = 3). ^∗^Statistical significance in comparison to the control group. ^∗^*p* < 0.05, ^∗∗^*p* < 0.01, and ^∗∗∗^*p* < 0.001. One-way ANOVA followed by Tukey's *post hoc* test.

To verify if Al and LPS could modulate the activity of purinergic system ectoenzymes, the breakdown of nucleotides and nucleoside was evaluated in microglial cells and the results are shown in Figure 2. NTPDase activity was assessed by ATP and ADP hydrolysis (Figures 2(a) and 2(b)). LPS was shown to significantly reduce the hydrolysis of ATP and ADP when compared to control cells. Moreover, the AlCl_3_ concentrations of 500 and 1000 *μ*M also reduced ATP hydrolysis, and AlCl_3_ at 1000 *μ*M also decreased ADP hydrolysis when compared to control cells. The activity of 5′-NT was assessed by AMP hydrolysis (Figure 2(c)), and results revealed that LPS and AlCl_3_ at 500 and 1000 *μ*M decreased AMP hydrolysis when compared to control cells. Following the ectoenzyme cascade, ADA activity (Figure 2(d)) was measured by the deamination of adenosine and findings revealed that LPS and AlCl_3_ at the concentrations of 500 and 1000 *μ*M augmented nucleoside breakdown compared to control cells.

### 3.3. Al and LPS Modulate the Expression and Density of Purinoceptors

**Figure 3:**
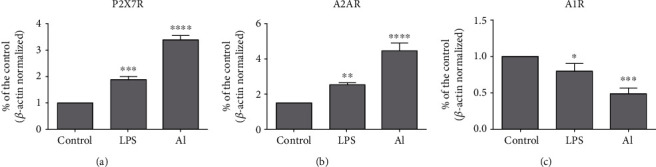
Gene expression of purinoceptors by RT-qPCR analysis of microglial cells treated with LPS (1 *μ*g/mL) and AlCl_3_ (1000 *μ*M) for 96 h. Al = aluminum; LPS = lipopolysaccharide. Values are expressed as mean ± SD (*n* = 3) of the relative concentration compared to the control group. ^∗^Statistical significance in comparison to the control group. ^∗^*p* < 0.05, ^∗∗^*p* < 0.01, ^∗∗∗^*p* < 0.001, and ^∗∗∗∗^*p* < 0.001. One-way ANOVA followed by Tukey's *post hoc* test.

**Figure 4:**
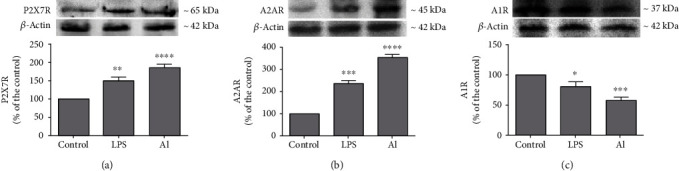
Protein density of purinoceptors by Western blot analysis of microglial cells treated with LPS (1 *μ*g/mL) and AlCl_3_ (1000 *μ*M) for 96 h. Al = aluminum; LPS = lipopolysaccharide. Values are expressed as mean ± SD (*n* = 3). ^∗^Statistical significance in comparison to the control group. ^∗^*p* < 0.05, ^∗∗^*p* < 0.01, ^∗∗∗^*p* < 0.001, and ^∗∗∗∗^*p* < 0.001. One-way ANOVA followed by Tukey's *post hoc* test.

Since Al and LPS modulated ectoenzyme activities, we also investigated whether these compounds could alter the expression and density of purinergic receptors.  Figure 3 shows the results from the analysis by RT-qPCR performed to assess the modulation of the P2X7R, A2AR, and A1R purinoceptor gene expression. Results showed that both LPS and Al significantly upregulated the expression of the P2X7R and A2AR purinoceptors whereas they downregulated the expression of the A1R purinoceptor when compared to the control group. Complementary to RT-qPCR findings, Western blot analysis also showed that LPS and Al were capable of modulating the protein levels of purinoceptors by significantly upregulating the density of the P2X7R and A2AR receptors and downregulating the density of the A1R receptor compared to the control group (Figure 4).

## 4. Discussion

Literature data have underlined the involvement of the purinergic system in the inflammatory responses and microglia behavior within the CNS [22, 24]. However, the impact of Al on the modulation of the purinergic system in microglial cells is poorly understood. In the current study, we showed that Al, and also LPS, disturbed some purinergic system parameters of BV-2 cells, evidenced by the changes in ectoenzymes activities and expression/density of purinoceptors.

Since Al^3+^, the neurotoxic and biologically reactive Al free ion, can pass the BBB and deposit in the brain tissue [6–9], microglial cells are within the reach of this element. Therefore, the possible uptake of Al was tracked qualitatively using Lumogallion fluorescent probe. This chemical has been proposed to generate an orange fluorescence signal when binding to the soluble Al^3+^ free ion [13, 31], as indicated in Figure 1(c)–1(i), leading to the suggestion that Al can be internalized by these immune cells.

Based on this assumption, it is relevant to comprehend the possible mechanisms related to the toxicity of the different stimuli used here in microglial cells. It is also noteworthy to comment that, although there are shortcomings of employing *in vitro* cellular models, the cell line used here has been proposed as a valuable substitute platform for *in vivo* microglia [38], suggesting these immune cells as a potential tool to investigate the effects triggered by the exposure to toxic agents.

Insults and noxious stimuli that alter CNS homeostasis, such as Al and LPS, may trigger the outpour of key purinergic messengers from microglial cells [24]. Changes in the levels of extracellular molecules, such as ATP and its breakdown products, are sensed by membrane-bound enzymes of the purinergic pathway, including NTPDase, 5′-NT, and ADA [14–17]. In this way, the possible changes in the activity of these key enzymes were further investigated. Similar to our findings, a previous investigation using blood lymphocytes of rats [39] also reported that LPS may affect the metabolism of nucleotides and nucleoside, reducing ATP hydrolysis and increasing adenosine breakdown. Moreover, ATP and AMP hydrolysis as well as NTPDase1 and 5′-NT expression have been shown diminished in a model of M1 macrophages (challenged with LPS) [40].

Concerning the effects observed for Al on enzyme activities, these could reflect the capacity of Al^3+^ to substitute metal ions and/or its interaction with nucleotides. The Al free ion may be able to displace native ions, such as Mg^2+^, at protein binding sites, thus being able to affect their biological functions [9, 41–43]. For instance, NTPDase is a metalloenzyme that requires Ca^2+^ or Mg^2+^ ions for its optimal activity and may have its functioning altered in the lack of these ions [15]. Moreover, the ability of Al^3+^ to interact with molecules, such as ATP [43], could reduce the availability of this nucleotide and interfere with the activity of membrane-bound enzymes of the purinergic pathway [13].

A coordinated upregulation of 5′-NT and purinoceptor expression, particularly A2AR expression, has been suggested by previous reports such as in hippocampal astrocytes of human patients with mesial temporal lobe epilepsy (MTLE) [44], in a rat model of Parkinson's disease [45], and in a rat model of AD [46]. However, although our results regarding the A2AR receptor are in agreement with these previous reports, the decrease in 5′-NT activity was also indicated when neurospheres were treated with Al^3+^ [13].

Also, an increase in ADA enzyme activity for cells treated with Al and LPS was found here. Adenosine, the main ATP breakdown product binds to P1 type of receptors, including A1R and A2AR subtypes of receptors, and together with ATP presents central neuromodulatory and immunomodulatory functions in the brain [19, 22, 47, 48]. Thus, the alterations evoked by LPS and Al regarding purinergic parameters could also influence immune/microglial responses in the brain, but these effects are issues to be addressed in more detail by further studies.

As microglial cells are recognized to express essentially all types of purinergic system proteins [22, 24], the effect of Al and LPS exposure on the modulation of purinoceptors could also be a relevant mechanism underlying the toxicity of these agents in neuroimmune cells. P2X7R receptors are ATP-gated ionotropic channels indicated to present a major role in neurodegeneration and neuroinflammation [49]. The increased expression of P2X7R receptors has also been suggested in microglial cells of A*β*-injected rat brains and human brains of AD patients [50], in microglia of a model using LPS administration [51], in immature rat brains exposed to lead (Pb) [52], and in the hippocampus of mouse pups also exposed to Pb [53]. In this sense, P2X7R receptors and downstream signaling pathways triggered by their activation may have a central contribution in the toxic mechanisms triggered by Al and LPS and offer the possibility for future exploration.

Adenosine signaling is also of particular relevance in the brain [47, 48]. For instance, an increased expression of the A2AR receptor has also been indicated for other brain insult models, for example, perinatal brain injury [54] and in LPS-treated microglial cells [55]. In our study, the expression/density of the A1R receptor were found decreased whereas A2AR receptor expression/density were increased. These findings are of significance considering that A1R receptors (inhibitory) have been mainly associated with neuroprotection whereas the A2AR receptors (facilitatory) to neurodegeneration and neuroinflammation. Moreover, the antagonism of A2AR receptors may also provide neuroprotection in several disease conditions [47, 48, 56–58]. However, inhibition of both adenosine receptors, A1R and A2AR, has also been suggested to afford neuroprotection against AlCl_3_ exposure in neuroblastoma cells [59]. Therefore, these outcomes and the findings of our study highlight that adenosine receptors are also candidates for further investigation.

Taken together, our findings suggested that microglial cells can uptake the Al^3+^ free ion, displayed by the orange fluorescence labeling tracked by Lumogallion reagent. The activities of ectoenzymes showed a decrease/increase in the metabolism of nucleotides/nucleoside, respectively, when cells were exposed to both stimuli. Moreover, Al and LPS upregulated the expression/density of purinoceptors generally associated with neurotoxicity and neuroinflammation and downregulated the expression/density of purinoceptors related to neuroprotection. In this sense, our current findings provide a possible link between Al and also LPS toxic effects and purinergic system alterations in microglial cells and support future studies to clarify these issues.

## 5. Conclusions

In summary, to the best of our knowledge, our results report some first indication on the possible involvement of the purinergic system in the mechanisms of Al toxicity in brain microglial cells. This agent evoked alterations in the setup of the purinergic system suggesting that this signaling pathway may be further investigated as a pivotal factor to understand the effects triggered by toxic compounds in the CNS.

## Data Availability

The data used to support the findings of this study are available from the corresponding author, upon reasonable request.

## Abbreviations

5′-NT: 5′-Nucleotidase
A1R: Adenosine A1 receptor
A2AR: Adenosine A2A receptor
AD: Alzheimer's disease
ADA: Adenosine deaminase
Ado: Adenosine
ADP: Adenosine diphosphate
Al: Aluminum
Al^3+^: Al trivalent ion
AlCl_3_: Aluminum chloride
AMP: Adenosine monophosphate
ANOVA: Analysis of variance
ATP: Adenosine triphosphate
BBB: Blood-brain barrier
CNS: Central nervous system
DAPI: 4′,6-Diamidino-2-phenylindole
FBS: Fetal bovine serum
LPS: Lipopolysaccharide
NPCs: Neural progenitor cells
NTPDase: Nucleoside triphosphate diphosphohydrolase
P1: Adenosine receptors
P2: ATP/ADP receptors
PFA: Paraformaldehyde
RT-qPCR: Real-time quantitative polymerase chain reaction
RIPA: Radioimmunoprecipitation assay
TBST: Tris-buffered saline with Tween
UDP: Uridine diphosphate
UTP: Uridine triphosphate

## Additional Points

*Highlights*. Aluminum (Al) and lipopolysaccharide (LPS) modulated purinergic system parameters of BV-2 brain microglial cells. Al and LPS altered the metabolism of nucleotides (ATP, ADP, and AMP) and nucleoside (adenosine). Al and LPS upregulated the expression/density of purinoceptors associated with neurodegeneration and neuroinflammation. Al and LPS downregulated the expression/density of purinoceptors associated with neuroprotection.
